# The negative aftermath of prostate biopsy: prophylaxis, complications and antimicrobial stewardship: results of the global prevalence study of infections in urology 2010–2019

**DOI:** 10.1007/s00345-021-03614-8

**Published:** 2021-02-22

**Authors:** Jakhongir F. Alidjanov, Tommaso Cai, Riccardo Bartoletti, Gernot Bonkat, Franck Bruyère, Béla Köves, Ekaterina Kulchavenya, José Medina-Polo, Kurt Naber, Tamara Perepanova, Adrian Pilatz, Zafer Tandogdu, Truls E. Bjerklund Johansen, Florian M. Wagenlehner

**Affiliations:** 1 Clinic for Urology, Pediatric Urology and Andrology, Justus-Liebig-University Giessen, Giessen, Germany; 2 Department of Urology, Santa Chiara Hospital, Trento, Italy; 3 Department of Urology, University of Pisa, Pisa, Italy; 4 Department of Urology, Alta Uro AG, Basel, Switzerland; 5 Urologie, CHU Bretonneau, Tours, France; 6 Université François Rabelais de Tours, PRES Centre Val de Loire, Tours, France; 7 Department of Urology, South-Pest Teaching Hospital, Budapest, Hungary; 8 Urogenital Department, Novosibirsk Research TB Institute, Koves Str 1. 1204, Budapest, 630040 Novosibirsk, Russian Federation; 9 Department of Urology, Hospital Universitario 12 de Octubre, Madrid, Spain; 10 School of Medicine, Technical University of Munich, Munich, Germany; 11 Department of Urinary Tract Infections and Clinical Pharmacology N.A, Lopatkin Scientific Research Institute of Urology and Interventional Radiology, Branch of the National Medical Research Radiological Centre of the Ministry of Health of the Russian Federation, Moscow, Russian Federation; 12 Department of Urology, University College London Hospitals, London, UK; 13 Department of Urology, Oslo University Hospital, Institute of Clinical Medicine, University of Oslo, Oslo, Norway; 14 Institute of Clinical Medicine, University of Aarhus, Aarhus, Denmark

**Keywords:** Prostate biopsy, Antibiotic resistance, Prostate cancer, Antibiotics, Fluoroquinolones

## Abstract

**Purpose:**

To evaluate and report the complications, and to analyse antimicrobial stewardship aspects following prostate biopsies (P-Bx) based on the data from a 9-year global study.

**Methods:**

The primary outcome was to compare complications after P-Bx between patients of two cohorts: 2010–2014 and 2016–2019. Primary outcomes included symptoms of lower and severe/systemic urinary tract infection (LUTIS and SUTIS, respectively), and positive urine culture. Readmission to hospital after P-Bx, need for additional antimicrobial therapy, consumption of different antimicrobial agents for prophylaxis and therapy were evaluated. Students *t* test and chi-square test were used for comparative analyses.

**Results:**

Outcome data were available for 1615 men. Fluoroquinolones-based prophylaxis rate increased from 72.0% in 2010–2014 to 78.6% in 2015–2019. Overall rates of complications increased from 6 to 11.7% including an increase in symptomatic complications from 4.7 to 10.2%, mainly due to an increase in LUTIS. Rates of patients seeking additional medical help in primary care after P-Bx increased from 7.4 to 14.4%; cases requiring post P-Bx antibiotic treatment increased from 6.1 to 9.7%, most of which received fluoroquinolones. Transperineal P-Bx was significantly associated with LUTIS. Following transrectal P-Bx, 2.8% developed febrile infections and 4.0% required hospitalisation. Two men (0.12%) died after transrectal P-Bx due to sepsis.

**Conclusions:**

The rates of complications after P-Bx tended to increase in time, as well as rates of patients seeking additional medical help in the post-P-Bx period. To reduce the risk of infectious complications and to comply with the principles of antibiotic stewardship, clinicians should switch to the transperineal biopsy route.

**Supplementary Information:**

The online version contains supplementary material available at 10.1007/s00345-021-03614-8.

## Introduction

Prostate biopsy (P-Bx) is one of the most commonly performed urological procedures worldwide, more than one million biopsies are performed annually in the United States alone [1]. Since introduction of transrectal biopsies, the symptomatic infectious complications rates have varied from 1.9 to 27.7% and have tended to increase [2–7]. Apart from the contamination category of the procedure, risk factors for post-biopsy infections remain unclear. However, in a recent systematic review, Borghesi et al. reported that the highest rates of infective complications were related to comorbidities and old age [6].

Results of the Global Prevalence Study of Infections in Urology (GPIU), a cross-sectional survey, initiated in 2003 by the board of the European Society for Infections in Urology (ESIU) showed that antimicrobial resistance (AMR) among common uropathogens increasingly lead to failure of antibiotic prophylaxis, thereby underlining the importance of site-specific antimicrobial stewardship programmes [8–11]. A side study on infectious complications of P-Bx in 2010 and 2011 reported that 5.2% of men experienced symptomatic urinary tract infection (UTI), 3.5% experienced febrile infectious complications and 3.1% required hospital re-admission after P-Bx [12].

The primary aim of the present study was to identify temporal trends in infective complications after P-Bx within the years 2010 to 2019 and compare two cohorts 2010–2014 and 2016–2019. Our secondary aim was to analyse antimicrobial stewardship aspects such as the percentage of patients seeking medical help of any kind and the rate of antibiotic treatment for infective complications after P-Bx.

## Methods

### Study design and setting

The GPIU P-Bx study is a prospective, observational online study, conducted annually in urology departments to audit the prevalence of infective complications after P-Bx across centres and countries and to evaluate factors associated with a higher risk of complications. Patients undergoing P-Bx within 2 weeks before the predefined study days each year were eligible for inclusion. Each enrolled patient was followed up for 2 weeks after the biopsy [12–14]. The severity of infectious complications was grouped according to the Center for Disease Control (CDC) criteria, in line with European Association of Urology (EAU) guidelines [15]. Ethical approval and regulatory issues were responsibility of each study centre [16].

Here, we cover the study years from 2010 to 2019, except for 2015 when the P-Bx study was not conducted due to updating of the online database. We, therefore, decided to use this year as a cut-off to allocate patients into two cohorts 2010–2014 and 2016–2019, respectively, depending on the year of registration.

### Patient information

The clinical report forms consisted of two parts. The first part included items on:

- Patient characteristics, such as age, use of antibiotics in the preceding 6 months, history of urinary tract infections (UTI) and history of antibiotic treatment in the preceding 6 months, prostate volume, prostate-specific antigen (PSA) value and previous P-Bx.
- Biospsy characteristics such as biopsy route, results of preoperative urine culture; if preoperative bowel preparation was performed and its type; if antibiotic prophylaxis was administered and if yes, which antimicrobial agent, the number of biopsy cores taken and use of local anaesthesia.

The second part of the study report form included:

- Clinical and microbiological outcome variables after the P-Bx such as presence and severity of symptoms of UTI at any time up to 2 weeks after P-Bx, (re)admission to hospital, results of post-P-Bx urine culture, type of antibiotics administered for treatment of infectious complications.
- Histological parameters such as presence and grade of histopathologic inflammation (low, moderate, severe).

### Data processing and statistical analysis

Symptoms were classified as lower UTI symptoms (LUTIS) such as frequency, dysuria, urgency and prostate pain, or symptoms of severe/systemic UTI (SUTIS), such as loin pain, rigour and fever. The majority of variables were categorical (presence/absence of symptom or characteristic) and, hence, dichotomized. Multiple imputations were performed on missing numerical values depending on the distribution, the median was used for the non-normally distributed and mean was used for normally distributed numerical variables. Missing categorical variables were not imputed to avoid biases.

Outcomes such as LUTIS, SUTIS and positive urine culture after P-Bx were assessed and compared both separately and in combination. Admission to hospital and need for antimicrobial treatment in the post-P-Bx period were considered as negative outcomes.

Continuous variables were presented in averages such as median and interquartile ranges (IQR) and compared between cohorts using two-sided Student *t* test with the Welch correction in cases of inequality of variances. Categorical variables were presented in proportions of the total study population and compared with the chi-square test concerning the total numbers of cases in cohorts. Statistical significance was set at 0.05. Statistical analysis and graphical representation of the results were performed using R-studio supporting the R-4.0.2 with in-built and additional packages [17–19].

## Results

Data from 258 clinics from 55 countries were considered valid for analysis (see Appendix). The number of participating centres went down from 174 in years 2010–2014 to 84 in years 2016–2019 (Table 1). The number of inputted valid cases was 2215. The range per study centre was 1–95 (median = 5, IQR = 2–10). Complete outcome data were available for 1615 cases (72.9% of total), all of which were included in further analysis. Of these, 1204 (74.6%) were included in years 2010–2014, and 411 (25.4%) in years 2016–2019 (Supplementary Fig. 1, Table 1).

**Table 1:** Demographics and outcomes in patients according to the total sample and cohorts

Parameter	Total	Cohort 2010–2014	Cohort 2016–2019	*P *value*
Demographics				
Number of participating centres, *n*	258	174	84	n.a
Number of cases, *n* (%)	1615 (100.0)	1204 (74.6)	411 (25.4)	n.a
Age, year, median (IQR)	66 (61.0–72.0)	66 (61.0–71.0)	67 (61.0–72.0)	0.675
Volume of prostate, ml, median (IQR)	46 (43.0–86.5)	46 (46.0–161.0)	46 (35.0–60.0)	< 0.001
PSA value, ng/ml, median (IQR)	9.0 (6.1–41.7)	10.4 (6.4–98.0)	8.0 (6.0–12.0)	< 0.001
Route of biopsy: perineal/transrectal, *n* (%)	56/1559 (3.5/96.5)	32/1172 (2.7/97.3)	24/387 (5.8/94.2)	0.004
Number of cores, median (IQR)	12 (10.0–12.0)	12 (10.0–12.0)	12 (10.0–12.0)	0.273
Repeated biopsy, *n* (%)	307 (19.0)	218 (18.1)	89 (21.7)	0.131
Number of previous biopsies, median (IQR)	1 (1.0–2.0)	1 (1.0–2.0)	1 (1.0–2.0)	0.458
History of UTIs, *n* (%)	132 (8.2)	102 (8.5)	30 (7.3)	0.514
Comorbid diabetes, *n* (%)	112 (6.9)	58 (4.8)	54 (13.1)	0.026
Presence of the urinary catheter, *n* (%)	73 (4.5)	44 (3.7)	29 (7.1)	0.138
Duration of the catheter stay, days, median (IQR)	10 (5.0–20.0)	10 (7.0–20.0)	6 (1.0–24.0)	0.544
AB therapy for any reason in preceding 6 months., *n* (%)	203 (12.6)	138 (11.5)	65 (15.8)	0.051
With fluoroquinolones, *n* (%)	114 (7.1)	75 (6.2)	39 (9.5)	0.055
With penicillins, *n* (%)	39 (2.4)	27 (2.2)	12 (2.9)	0.636
With TMP-SMX, *n* (%)	20 (1.2)	15 (1.2)	5 (1.2)	1.000
With cephalosporins, *n* (%)	14 (0.9)	11 (0.9)	3 (0.7)	0.918
With other antimicrobial agents, *n* (%)	16 (1.0)	10 (0.8)	6 (1.5)	0.410
Duration of the AB therapy in preceding 6 months, days, median (IQR)	7 (7.0–14.0)	7 (5.0–10.5)	7 (7.0–14.0)	0.027
Positive urine culture before P-Bx, *n* (%)	27 (1.7)	17 (1.4)	10 (2.4)	0.645
AB prophylaxis before P-Bx, *n* (%)	1503 (93.1)	1109 (92.1)	394 (95.9)	0.013
With fluoroquinolones, *n* (%)	1190 (73.7)	867 (72.0)	323 (78.6)	0.011
With combinations of ABs, *n* (%)	137 (8.5)	129 (10.7)	8 (1.9)	< 0.001
With cephalosporins, *n* (%)	56 (3.5)	29 (2.4)	27 (6.6)	< 0.001
With aminoglycosides, *n* (%)	52 (3.2)	31 (2.6)	21 (5.1)	0.019
With penicillins, *n* (%)	17 (1.1)	10 (0.8)	7 (1.7)	0.224
With TMP-SMX, *n* (%)	15 (0.9)	10 (0.8)	5 (1.2)	0.684
With oxacephems, *n* (%)	15 (0.9)	15 (1.2)	0 (0.0)	0.048
With other antimicrobial agents, *n* (%)	21 (1.3)	18 (1.5)	3 (0.7)	0.352
Duration of the AB prophylaxis, days, median (IQR)	3 (1.0–5.0)	3 (1.0–5.0)	2 (1.0–4.0)	0.014
Bowel preparation before P-Bx, *n* (%)	570 (35.3)	408 (33.9)	162 (39.4)	0.057
Enema, *n* (%)	444 (27.5)	317 (26.3)	127 (30.9)	0.094
Lavage, *n* (%)	32 (2.0)	24 (2.0)	8 (1.9)	1.000
Other, *n* (%)	94 (5.8)	67 (5.6)	27 (6.6)	0.544
Anaesthesia, *n* (%)	1099 (68.0)	801 (66.5)	298 (72.5)	0.055
Local, *n* (%)	985 (61.0)	721 (59.9)	264 (64.2)	0.204
General, *n* (%)	97 (6.0)	69 (5.7)	28 (6.8)	0.531
Spinal, *n* (%)	17 (1.1)	11 (0.9)	6 (1.5)	0.525
Outcomes				
Histopathologic signs of inflammation in the prostatic tissue, *n* (%)	455 (28.2)	321 (26.7)	134 (32.6)	0.025
Mild, *n* (%)	280 (17.3)	214 (17.8)	66 (16.1)	0.545
Moderate, *n* (%)	141 (8.7)	83 (6.9)	58 (14.1)	< 0.001
Severe, *n* (%)	34 (2.1)	24 (2.0)	10 (2.4)	0.736
Cases with at least 1 negative outcome, *n* (%)	122 (7.6)	74 (6.1)	48 (11.7)	< 0.001
Symptomatic cases	111 (6.9)	64 (5.3)	47 (11.4)	< 0.001
LUTIS, *n* (%)	98 (6.1)	56 (4.7)	42 (10.2)	< 0.001
Dysuria, *n* (%)	75 (4.6)	42 (3.5)	33 (8.0)	< 0.001
Frequency, *n* (%)	47 (2.9)	29 (2.4)	18 (4.4)	0.060
Urgency, *n* (%)	38 (2.4)	24 (2.0)	14 (3.4)	0.149
Prostate pain, *n* (%)	32 (2.0)	17 (1.4)	15 (3.6)	0.009
Number of LUTIS per case, median (IQR)	2 (1.0–3-0)	2 (1.0–3.0)	1 (1.0–3.0)	0.651
Symptoms of UTI, *n* (%)	46 (2.8)	33 (2.7)	13 (3.2)	0.785
Loin pain, *n* (%)	4 (0.2)	3 (0.2)	1 (0.2)	1.000
Rigour, *n* (%)	6 (0.4)	1 (0.1)	5 (1.2)	0.005
Fever, *n* (%)	42 (2.6)	31 (2.6)	11 (2.7)	1.000
Number of UTI symptoms per case, median (IQR)	1 (1.0–1.0)	1 (1.0–1.0)	1 (1.0–2.0)	0.025
Number of symptoms (LUTIS and UTI) per case, median (IQR)	2 (1.0–3.0)	2 (1.0–3.0)	2 (1.0–3.0)	0.353
Positive urine culture after biopsy, *n* (%)	39 (2.4)	28 (2.3)	11 (2.7)	1.000
Symptomatic cases with positive urine culture after biopsy, *n* (%)	28 (1.7)	18 (1.5)	10 (2.4)	0.265
Isolated *E. coli*, *n* (%)	29 (1.8)	20 (1.7)	9 (2.2)	0.577
Resistance to antimicrobials, *n* (%)	103 (6.4)	51 (4.2)	52 (12.7)	< 0.001
Cephalosporins, *n* (%)	25 (1.5)	12 (1.0)	13 (3.2)	0.030
Penicillins, *n* (%)	19 (1.2)	8 (0.7)	11 (2.7)	0.059
Aminoglycosides, *n* (%)	12 (0.7)	5 (0.4)	7 (1.7)	0.050
Fluoroquinolones, *n* (%)	19 (1.2)	16 (1.3)	3 (0.2)	0.185
TMP-SMX, *n* (%)	9 (0.6)	6 (0.5)	3 (0.7)	1.000
Other classes of antimicrobials, *n* (%)	19 (1.2)	4 (0.3)	15 (3.6)	< 0.001
Patients, seeking for medical help after P-Bx, *n* (%)	185 (11.5)	114 (9.5)	71 (17.3)	< 0.001
At primary care clinician's, *n* (%)	148 (9.2)	89 (7.4)	59 (14.4)	< 0.001
At emergency room, *n* (%)	37 (2.3)	25 (2.1)	12 (2.9)	0.426
Patients, required hospitalisation, *n* (%)	60 (3.7)	46 (3.8)	14 (3.4)	0.816
To urology ward, *n* (%)	52 (3.2)	41 (3.4)	11 (2.7)	0.575
To internal medicine ward, *n* (%)	6 (0.4)	3 (0.2)	3 (0.7)	0.361
To intensive care ward, *n* (%)	2 (0.1)	2 (0.2)	0 (0.0)	0.988
Antibacterial therapy, prescribed after P-Bx, *n* (%)	113 (7.0)	73 (6.1)	40 (9.7)	0.016
With fluoroquinolones, *n* (%)	54 (3.3)	33 (2.7)	21 (5.1)	0.032
With cephalosporins, *n* (%)	23 (1.4)	15 (1.2)	15 (1.9)	0.427
With aminoglycosides, *n* (%)	8 (0.5)	5 (0.4)	3 (0.7)	0.706
With penicillins, *n* (%)	7 (0.4)	5 (0.4)	2 (0.5)	1.000
With combination of antimicrobial agents, *n* (%)	7 (0.4)	5 (0.4)	2 (0.5)	1.000
With other antimicrobial agents, *n* (%)	14 (0.9)	10 (0.8)	4 (1.0)	1.000
Duration of the antibacterial therapy, days, median (IQR)	5 (5.0–10.0)	7 (5.0–8.5)	5 (5.0–10.0)	0.937
Resolved cases, *n* (%)	1458 (90.3)	1114 (95.0)	314 (76.4)	< 0.001
Unresolved cases, *n* (%)	66 (4.1)	53 (4.4)	13 (3.2)	0.342
Lethal cases, *n* (%)	2 (0.1)	1 (0.1)	1 (0.2)	1.000

*Cohort 2010–2014 vs. cohort 2016–2019

### Total population

#### Demographics

Median (IQR) age of patients included in the analysis was 66 (61.0–72.0) years. Of them, 307 (19.0%) underwent repeat biopsy. The median (IQR) number of previous biopsies was 1 (1.0–2.0).

A history of UTI was noted in 132 (8.2%) patients, comorbid diabetes in 112 (6.9%) and 73 (4.5%) patients had a urinary catheter at time of biopsy.

A history of previous antimicrobial treatment was positive in 203 (12.6%) patients, with fluoroquinolones being the most widely prescribed antimicrobial in 114 (7.1% of total).

Transrectal P-Bx was performed in 1559 (96.5%) patients and 56 (3.5%) underwent transperineal P-Bx.

Positive urine culture before P-Bx was noted in 27 (1.7%) cases (Table 1). Antimicrobial prophylaxis before P-Bx was reported in 1503 (93.1%) patients, with fluoroquinolones as the most frequently prescribed class in 1190 (73.7%) (Table 1).

#### Clinical outcomes

**Fig. 1:**
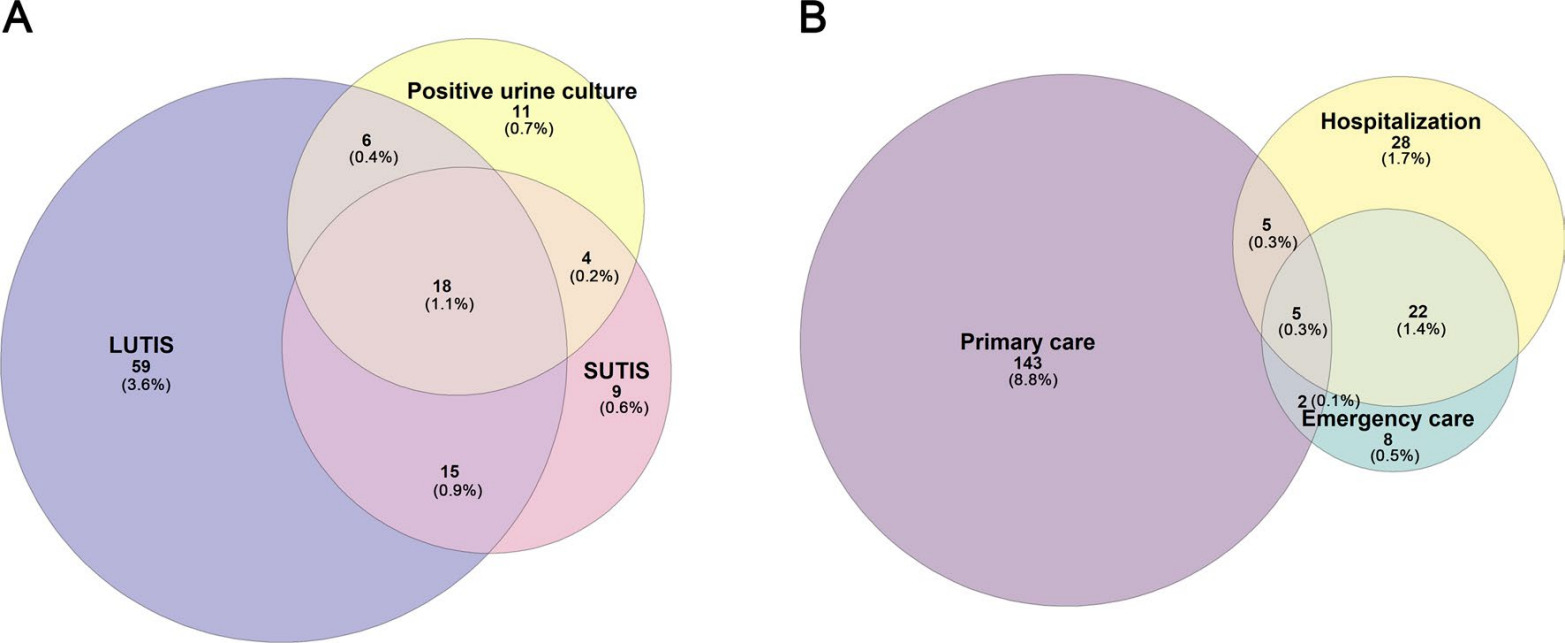
**a** Proportions of negative outcomes after P-Bx. **b** Proportions of cases requiring medical assistance after P-Bx

LUTIS were recorded in 98 (6.1%) patients, of which the most common single symptom was dysuria which was observed in 75 (4.6%) patients, and SUTIS was noted in 46 (2.8%), of which 42 cases (2.6%) had fever (Fig. 1a).

The proportion of cases with LUTIS was significantly (*p* < 0.001) higher in the group of patients who underwent perineal P-Bx (17.9%) than in those, who underwent transrectal P-Bx (5.6%). The proportion of cases with SUTIS was 3.0% among patients who underwent transrectal P-Bx. No case of SUTIS was recorded among the patients after perineal P-Bx.

Antimicrobial treatment was prescribed to 113 (7.0%) patients after P-Bx. Fever was the single symptom that most often led to treatment with antimicrobial agents (88.1%), followed by prostate pain (71.9%) and rigour (66.7%) (Supplementary Fig. 2). 185 (11.5%) patients were seeking additional medical help, and 60 patients (3.7%) were hospitalised due to complications (Fig. 1b). All hospitalised cases belonged to patients who underwent transrectal P-Bx.

In 66 (4.1%) patient’s complications had not resolved within the 2 week follow-up after P-Bx, and 2 (0.1%) lethal cases were noted due to urosepsis after transrectal P-Bx (Table 1).

#### Microbiological outcomes

Thirty-nine of 103 tested cases (2.4% of total) had a positive urine culture after P-Bx (Table 1, Fig. 1a). All cases of positive urine culture were recorded in the group of patients who underwent transrectal P-Bx. A single uropathogen was found in 36 (2.2%) cases, and a mixed flora was found in 3 cases (0.2%). *Escherichia coli* was the most commonly isolated uropathogen and was noted in 29 (1.8%) cases, followed by *Enterococci* (*n* = 3) and coagulase-negative *Staphylococci* (*n* = 3) (0.2% per case/sample) of cases, *Enterobacter *spp*.* (*n* = 2), and *Klebsiella *spp*.* (*n* = 2) (0.1% per case/sample). *Proteus* and *Pseudomonas* species were found in two cases (< 0.1% per case/sample). The highest resistance rates of uropathogens were found against cephalosporins (*n* = 25), penicillins (*n* = 19), fluoroquinolones (*n* = 19), and aminoglycosides (*n* = 12).

### Comparison between cohorts and trends over time

#### Demographics

The average age of the patients did not differ significantly between cohorts, neither did the proportions of repeat biopsy, history of UTI and antimicrobial treatment, positive urine culture before biopsy, presence of urinary catheter or duration of catheterisation. The proportion of patients with diabetes mellitus was significantly higher in cohort 2016–2019 (Table 1).

#### Biopsy-related variables

The preparation and performance of P-Bx (bowel preparation, anaesthesia, number of biopsy cores) did also not differ. However, the route of P-Bx showed a statistically significant increase in the rates of transperineal P-Bx with a subsequent significant decrease in the number of transrectal P-Bx with 32 and 1172 (2.7 and 97.3%) cases in 2010–2014 vs 24 and 387 (5.8 and 94.2%) cases in 2016–2019 for transperineal and transrectal routes, respectively (*p* < 0.001).

#### Symptoms

Overall rates of complications increased from 6.1% in the cohort of 2010–2014 to 11.7% in the 2016–2019 cohort (*p* < 0.001) (Supplementary Fig. 1, Table 1).

The proportion of LUTIS, dysuria and prostate pain was significantly higher in the cohort of 2016–2019 (*p* < 0.01). The overall rates of SUTIS remained the same between cohorts; while, the rates of rigour as single symptom were significantly higher in the cohort of 2016–2019, as well as the average number of SUTIS (*p* < 0.05).

The proportions of cases which resolved within 2 weeks after P-Bx decreased significantly from 95.0 to 76.4% from the period 2010–2014 to 2016–2019 (*p* < 0.001) (Table 1).

#### Microbiological variables

Among patients with a history of antibiotic treatment before P-Bx, the duration of treatment was significantly longer in the cohort 2016–2019 (*p* = 0.027). The proportion of cases receiving antimicrobial prophylaxis (especially with fluoroquinolones, cephalosporins, and aminoglycosides) was significantly higher in the cohort 2016–2019; whereas, average duration of prophylaxis was significantly lower (*p* < 0.05) (Table 1).

The resistance of uropathogens was significantly higher in the cohort 2016–2019 particularly due to resistance against cephalosporins (*p* < 0.05) (Table 1).

#### Need for medical assistance

The proportions of patients seeking medical help within 2 weeks after P-Bx increased in 2016–19, mainly among patients who sought their GP`s office (*p* < 0.001); whereas, the numbers of patients who needed hospitalisation did not differ significantly between cohorts (*p* = 0.816). Prescription of antimicrobial therapy after P-Bx increased from 6.1 to 9.7%, with a significant increase in the prescription of fluoroquinolones (*p* < 0.05).

## Discussion

Infectious complications occur in 5–7% of all transrectal P-Bx. Severe infections requiring hospitalisation are seen after 1–3% of biopsies and fatal events are reported in 0.1–1.3% [20]. During the recent years, there has been a rise in infectious complications after P-Bx, thought to be due to increasing antibiotic resistance, especially to fluoroquinolones which have been recommended by the European and the American Association of Urology Guidelines as the first-choice antibiotic prophylaxis in P-Bx in the past [15, 21]. The antibiotic stewardship perspectives of the extensive use of fluoroquinolones have been questioned [1, 21].

In this study, we could demonstrate a significant global increase in the rate of infective complications after transrectal P-Bx from 2010 to 2019. We found a twofold increase in the rate of symptoms from 5.3% in the cohort of 2010–2014 to 11.4% in the cohort of 2016–2019, paralleled by an increase in the rate of dysuria, prostate pain (LUTIS), rigour (a symptom of SUTIS) and an average number of symptoms per case. Moreover, there were two cases of death due to septic shock after transrectal P-Bx (0.1%), a rate that equals house mortality after radical prostatectomy [22]. The number of patients who sought medical help for any complication and the number of patients who received antibiotic treatment for infective complications increased significantly in the most recent cohort. Also, the number of resolved cases within two weeks decreased from 95.0% in 2010–2014 to 76.4% in 2016–2019.

The use of fluoroquinolones as prophylactic agents increased during study years and remain the most commonly prescribed agents for P-Bx prophylaxis worldwide. In the first analysis of the GPIU P-Bx study, fluoroquinolones were used for prophylaxis in 98.2% of patients and 60% of all bacterial strains isolated after the procedure were resistant to this drug [12]. Several authors have shown that *E. coli* is the most common pathogen in terms of infective complications after P-Bx [23]. Moreover, prebiopsy rectal cultures have demonstrated a fluoroquinolone-resistant colonisation rate of 10–22% [20]. We did not find a significant difference in rates of fluoroquinolone-resistant strains between cohorts, but there was an almost threefold increase in overall resistance rates of uropathogens against antimicrobial agents from 4.2 to 12.7%. We also found a significant increase in the overall prescription of antimicrobial prophylaxis for almost all classes of antimicrobial agents, including fluoroquinolones and cephalosporins (72.0 vs. 78.6, and 2.4 vs. 6.6% for 2010–2014 vs. 2016–2019, respectively). Patients with a history of preceding antimicrobial treatment were at higher risk of developing complications after transrectal P-Bx. Steensels and co-authors demonstrated that the use of fluoroquinolones 6 months before P-Bx was associated with an increased risk of faecal carriage of fluoroquinolone-resistant *E. coli* strains [24]. Our findings demonstrate that the rate of symptomatic infective complications after transrectal P-Bx is high and is associated with antimicrobial resistance and use of antibiotics both as prophylaxis and as treatment of complications. This violates the principles of antimicrobial stewardship and increases health care costs.

Scott et al. showed that in cases of empirical antimicrobial prophylaxis without prior urine culture, the infection rate was 3.4% (95% CI 2.6–4.3%). In cases of culture-based targeted antibiotic prophylaxis, the infection rate was 0.8% (95% CI 0.4–1.3%) [23]. In our first GPIU P-Bx study, we argued that the rate of systemic infection was higher than earlier reports due to GPIU recruiting patients from a global average of urology departments and not from selected centres only. Other recent publications also demonstrate an increase in negative outcomes after P-Bx, including infectious complications [25–27]. In this study, we included all symptomatic complications, not only infections confirmed by microbiological culture. In the GPIU protocol, we do request microbiological analysis, but this was performed in only 103 cases (6.4%). By separating symptoms to SUTIS and LUTIS, respectively, we could demonstrate that SUTIS such as loin pain, rigour and fever were associated with transrectal P-Bx and not with transperineal P-Bx. The transrectal biopsy was associated with almost all remaining predefined complications (outcomes), such as positive urine culture after P-Bx and need for hospitalisation within the 2 weeks after P-Bx. Moreover, both lethal cases due to septic shock occurred after transrectal P-Bx. Our analysis demonstrated that patients who underwent transrectal P-Bx more often developed SUTIS, while patients with transperineal P-Bx were more likely to develop prostate pain and LUTIS.

Several clinical factors are associated with a higher risk of symptomatic complications [1]. In the present study, we demonstrate that transrectal P-Bx itself is a risk factor for complications and need for hospitalisation; while, the transperineal route is associated with LUTIS only. We believe this underlines that the contamination category of the biopsy procedure is the most significant risk factor for infective complications.

Fluoroquinolones, which have remained the most commonly used drugs in urological practice, were recently suspended for P-Bx prophylaxis by the European Medical Agency (EMA) [28]. Alternative antibiotic regimens are, therefore, warranted. A meta-analysis including only randomised studies exhibited limited evidence for using aminoglycosides, cephalosporins and fosfomycin trometamol for transrectal P-Bx prophylaxis [27]. Our findings support this view. We could, however, show that the use of aminoglycosides as prophylaxis was associated with a higher risk of symptomatic complications and a higher need for antibiotic therapy in the follow-up period after transrectal P-Bx. Detailed knowledge of local resistance data must, therefore, be taken into account in antibiotic prophylaxis protocols [29]. Our data suggest that to reduce patients’ risk of infectious complications and to comply with the principles of antibiotic stewardship, transperineal P-Bx should be prioritised [26, 30].

### Strengths and limitations

The worldwide, multicentric, multinational and prospective design are strengths of this study. The long enrolment period allows for detecting changes in the rates of infective complications and bacterial resistance over time.

Limitations to consider are the few centres per country, which means that data are not representative for each country. The low number of microbiological cultures after the procedure might be considered a study limitation. We argue, however, that our study reports the real-life situation in all centres.

## Conclusions

We emphasise the worldwide increase in complication rates (up to 11.4%) and the average number of symptoms such as dysuria, prostate pain and rigour after P-Bx, traceable from 2010 to 2019 in the GPIU study. The rate of fluoroquinolones prescribed as prophylaxis before, and as antimicrobial treatment after P-Bx also increased significantly. Transperineal P-Bx itself was a risk factor for development of LUTIS; whereas, transrectal P-Bx was associated with a wider spectrum of post-P-Bx complications including LUTIS and SUTIS, positive urine culture after the biopsy and risk of hospitalisation. The most severe infectious complications like febrile UTI and mortality were only seen after transrectal P-Bx.

## Supplementary Information

Below is the link to the electronic supplementary material.

Supplementary file1 Appendix Clinics and names of representing researchers, participating in the GPIU-Prostate biopsy studies 2010-2019, ordered alphabetically according to country names (DOCX 38 KB)

Supplementary file2 Supplementary Figure 1. Flow chart of inclusion and outcomes (TIF 321 KB)

Supplementary file3 Supplementary Figure 2. Proportions of symptoms leading to antimicrobial treatment within 2 weeks after P-Bx (PNG 109 KB)
