# Supplementary material for: The negative aftermath of prostate biopsy: prophylaxis, complications and antimicrobial stewardship: results of the global prevalence study of infections in urology 2010–2019
Source: World J Urol. 2021 Feb 22;39(9):3423–32. doi: 10.1007/s00345-021-03614-8 (PMC8510929; doi:10.1007/s00345-021-03614-8)
Supplement: Supplementary file 1 — Supplementary file1 Appendix Clinics and names of representing researchers, participating in the GPIU-Prostate biopsy studies 2010-2019, ordered alphabetically according to country names (DOCX 38 KB) [file 345_2021_3614_MOESM1_ESM.docx]

| **Country** | **City** | **Institution** | **Department** | **Representative researcher** | | |
| --- | --- | --- | --- | --- | --- | --- |
|  |  |  |  | **First name** | **Middle name** | **Last Name** |
| Algeria | Algiers | Chu Beni Messous | Isaad Hassani | Zidelkheir |  | Mohamed |
|  |  | Hôpital kouba | Urology | Mohsen |  | Azli |
|  | Oran | Centre d'urologie Oran | Oran | Nordine | M. | Benhatchi |
| Australia | Canberra | Calvary Bruce Public Hospital | Urology | Hin | Fan | Chan |
| Austria | Baden | Landesklinikum Baden | Urology | Ortwin |  | Heißler |
|  | Klagenfurt am Wörthersee | Klinikum Klagenfurt am Wörthersee | Urology | Tanja |  | Gschliesser |
| Belgium | Bonheiden | Imelda ziekenhuis | Antwerpen | Manu |  | Joris |
|  | Oostende | Az Damiaan | Urology | Pieter |  | D'hulst |
|  | Yvoir | CHU UCL Mont-Godinne | Urology | Michael |  | Twahirwa |
| Bosnia And Herzegovina | Sarajevo | University Clinical Centre Sarajevo | Urology Clinic | Senad |  | Bajramovic |
|  |  |  |  | Spahovic |  | Hajrudin |
|  |  |  |  | Hajrudin |  | Spahovic |
| Brazil | Florianopolis | CEPON Centro de Pesquisas Oncologicas | Service of Urology | Flávio | Lobo | Heldwein |
|  | Ijui | Hospital Unimed Ijuí | Centro Avançado de Urologia | Marcio | El Ammar | Muller |
| Cameroon | Yaounde | Central Hospital of Yaounde Cameroon | Urology | Jean Cédrick |  | Fouda |
| Croatia | Rijeka | University Hospital Rijeka | Urology | Dean |  | Markić |
|  | Varazdin | General hospital Varazdin | Urology | Branimir |  | Lodeta |
|  | Zagreb | Sestre milosrdnice University Hospital centre | Urology | Sven |  | Nikles |
|  |  |  |  | Matea |  | Pirša |
|  |  |  |  | Leo |  | Dumbovic |
|  |  | University Hospital Center Zagreb | Urology | Luka |  | Penezić |
|  |  | University Hospital Dubrava | Urology | Katarina |  | Grbesa |
|  |  | University Hospital Sveti Duh | Urology | Radmila |  | Vrbat |
| Czech Republic | Brno | Masarykův onkologický ústav | Oddělení urologické onkologie | Daniel |  | Macík |
|  | Liberec | Regional Hospital in Liberec | Urology | Jiří |  | Pírek |
|  | Praha | Thomayer Hospital | Urology | Jan |  | Hrbacek |
| Denmark | Viborg | Regionshospitalet Viborg | Urinvejskirurgi | Imre |  | Bode |
| Egypt | Alexandria | Alexandria University hospital | Urology | Omar | Farid | Elgebaly |
|  | Cairo | Ain Shams University Hospitals | Urology | Mohamed | Essam Eldin | Mohamed |
|  |  | National cancer institute | Oncourology | Abdelmaksoud | Mohamed | Ali |
|  | Elmehala Elkubra | Tarekelmorsy Hospital | Urology | Tarek Elmorsy | El Morsy | Aboaly |
|  | Zagazig | Zagazig University Hospital | Urology | Ahmed | Ragab | Ali |
| Estonia | Tallinn | North Estonia Medical Centre | General and oncological urology | Margus |  | Krabi |
|  | Tartu | Tartu University Hospital | Department of Urology and Kidney Transplantation | Mihhail |  | Žarkovski |
| Finland | Vantaa | Helsinki University Hospital | Urology | Henrikki |  | Santti |
| France | Tours | CHU Tours | Urology | Franck |  | Bruyere |
| Germany | Bielefeld | Franziskus Hospital | Urology | Andrei |  | Neculoiu |
|  | Brandenburg an der Havel | Städtisches Klinikum Brandenburg GmbH | Klinik für Urologie | Frank | Ronald | Benzing |
|  | Brilon | Urological Clinic Hochsauerland | Urology | Lucas | Goncalves | Prado |
|  | Freiburg | University Medical Center Freiburg | Urology | Daniel |  | Schlager |
|  | Gelsenkirchen | Bergmannsheil-Buer | Urology | Stephan |  | Miller |
|  | Giessen | University Hospital Gießen and Marburg | Clinic for Urology, Pediatric Urology and Andrology | Florian |  | Wagenlehner |
|  |  |  |  | Mareike |  | Buch-Heberling |
|  | Mönchengladbach | Kliniken Maria Hilf GmbH | Klink für Urologie | Joachim |  | Hirschmann |
|  | Reklinghausen | Prosper Hospital Recklinghausen | Urology | Philipp |  | Ganssmann |
|  | Rosenheim | Romed Klinikum Rosenheim | Urology | Tanja |  | Frank |
|  | Villingen-Schwenningen | Schwarzwald Baar Klinikum Villingen-Schwenningen | Abteilung für Urologie und Kinderurologie | Julius |  | von Süßkind-Schwendi |
| Ghana | Tamale - Ghana | Tamale Teaching Hospital | Urology | Akis | Alekz | Afoko |
| Greece | Arta | General Hospital of Arta | Urology | Nikolaos | Vasilios | Manolakis |
|  | Athens | Central Clinic of Athens | Urology | George |  | Karamichalis |
|  |  | General Hospital of Athens G.Gennimatas | Urology | Stavros | Konstantinos | Lamprou |
|  | Eghion | General Hospital of Eastern Achaia | Urology | Theodore |  | Voudoukis |
|  | Heraklion | University General Hospital Heraklion | Urology | Georgios |  | Georgiadis |
|  | Piraeus | Tzaneio Hospital | Urology | Konstantinos |  | Stamatiou |
|  |  |  |  | Evangelia |  | Samara |
|  | Thessaloniki | G.Gennimatas General Hospital of Thessaloniki | Urological Dept. of Aristotle University of Thessaloniki | Ioannis |  | Vakalopoulos |
|  |  | General Hospital of Thessaloniki "G. Gennimatas" | First Urology Clinic of the Aristotle University of Thessaloniki | Aikaterini |  | Tsionga |
|  |  | General Hospital of Thessaloniki Agios Pavlos | Urology | Vasileios | I. | Sakalis |
| Guernsey | St Andrews | Princess Elizabeth Hospital | Urology | Owen | James | Cole |
| Hungary | Budapest | Péterfy Sándor utcai Kórház | Urológia-sebészeti Osztály | Istvan |  | Buzogany |
|  |  | Buda Hospital of the Hospitaller Order of Saint John of God | Urology | Adam |  | Fukasz |
|  |  | Jahn Ferenc South-Pest | Urology | András |  | Magyar |
|  |  | Semmelweis University Urological Clinic | Department of Urology and Centre for Urooncology | János |  | Szalontai |
|  |  | National Institute of Oncology | Uro-Oncology | Ágnes |  | Rosecker |
|  | Pécs | University of Pécs, Urology Clinic | Urology Clinic | Lehel |  | Péterfi |
|  | Szeged | University of Szeged, Faculty of Medicine | Urology | Márton |  | Oroszi |
|  | Szombathely | Teaching Hospital Markusovszky | Urology | Levente |  | László |
| India | Ahmedabad | Civil Hospital | Urology | Shrenik | J. | Shah |
|  | Chennai | Chettinad health city | Urology | Bala | Ravi teja | Sepuri |
|  | Dhule | Institute of Urology | Urology | Sergii | Petrovich | Pasiechnikov |
|  |  | Kasturba medical college | Department of urology and renal transplantation | Avinash | R. | Odugoudar |
|  |  |  |  | Milap |  | Shah |
|  | Shimla | Indira Gandhi Medical College | Urology | Manjeet |  | Kumar |
|  | Vadodara | Kamat kidney and eye hospital | Urology | Nagesh | N. | Kamat |
|  | Wardha | Acharya Vinobha Bhave Rural Hospital | Urology | Abhijit | Shridhar | Dhale |
| Indonesia | Surabaya | Dr Soetomo General Hospital | Department of Urology, Dr Soetomo General Hospital, Faculty of Medicine Universitas Airlangga, Surabaya, Indonesia | Duje |  | Rako |
|  |  |  |  | Niwanda |  | Yogiswara |
| Iraq | Basrah | Basrah Teaching Hospital | Urology | Murtadha |  | Almusafer |
|  | Hilla | Hilla teaching hospital | Urology | Wadhah |  | Almarzooq |
| Islamic Republic of Iran | Ahvaz | Imam Khomeini | Urology | Dinyar |  | Khazaeli |
|  | Sanandaj | Kowsar | Urology | Nader | Rash | Ahmadi |
| Italy | Piombino (LI) | Ospedale Villamarina | Urology | Michele |  | Lanciotti |
|  | Alessandria | Azienda ospedaliera SS Antonio e Biagio Alessandria | Urology | Barbara |  | Cavallone |
|  | Cefalù | Ospedale G.Giglio | Unità operativa complessa Urologia | Tiziana |  | Puglisi |
|  | Guastalla | Civil Hospital of Guastalla, AUSL-IRCCS of Reggio Emilia | Urology Unit | Davide |  | Campobasso |
|  | Melegnano | ASST Melegnano Martesana Ospedale Vizzolo Predabissi | Unità Operativa Complessa Urologia | Sabato |  | Barra |
|  | Naples | AORN Antonio Cardarelli | Urology | Francesco |  | Chiancone |
|  | Negrar (VR) | Sacred Heart Hospital | Urology | Alberto |  | Molinari |
|  | Pisa | Azienda ospedaliera universitaria pisana | Urology | Ramona |  | Baldesi |
|  | Prato | Santo Stefano | Urology | Arben |  | Belba |
|  | Roma | Agostino Gemelli Universitary Hospital | Urologic Clinic | Emilio |  | Sacco |
|  |  |  |  | Mauro |  | Ragonese |
|  |  |  |  | Giuseppe |  | Palermo |
|  | San Fermo della Battaglia | ASST Lariana - Ospedale Sant'Anna | Surgery | Carmen |  | Maccagnano |
|  | San Giovanni in Persiceto | ospedale ss salvatore | Dipartimento di Chirurgia | Francesco |  | Andrei |
| Japan | Kitakyushu | Hospital of the University of Occupational and Environmental Health, Japan | Urology | Masahiro |  | Matsumoto |
|  | Sapporo | Sapporo Medical University Hosipital | Urology | Yoshiki |  | Hiyama |
| Jordan | Amman | Albashir Hospital | Urology | Ahmad |  | Sa'eed |
| Kuwait | Ahmadi | Ahmadi hospital (KOC) | Urology | Hasan |  | Motairy |
|  |  |  |  | Hani | Elsayed | Shaaban |
| Lithuania | Kaunas | Lithuanian University of Health Sciences | Urology | Zilvinas |  | Venclovas |
|  | Utena | Utena hospital | Surgery clinic, urological unit | Vitalijus |  | Usovas |
| Macedonia, The Former Republic Of Yugoslav | Skopje | University Clinic of Surgery" St. Naum Ohridski" Skopje | Urology | Slobodan | Petar | Ristovski |
|  |  |  |  | Maja | Sofronievska | Glavinov |
| Malaysia | Melaka | Oriental Melaka Straits Medical Centre | Urology | Christopher | Chee Kong | Ho |
| Mexico | Guadalajara | Centro de Urologia Integral | Urology | Godofredo |  | Neyra |
|  | Mexico city | Hospital General Dr. Manuel Gea González | Urology | Javier | Antonio | Herrera Muñoz |
|  | Puebla | IMSS Hospital de Especialidades Puebla | Urology | Carlos | Eleazar | Lopez |
| Morocco | Casablanca | CHU Ibn Rochd | Aile 5 | Chakir |  | Youness |
|  | Rabat | CHU Ibn Sina | Urology | Amine |  | Saouli |
| Netherlands | Amsterdam | Antoni van Leeuwenhoek Ziekenhuis | Urology | Bart |  | Geboers |
|  | Nieuwegein | St. Antonius Ziekenhuis | Urology | Sandrine |  | van Selm |
| Nigeria | Irrua | Irrua Specialist Teaching Hospital, Irrua | Surgery | Eshiobo |  | Irekpita |
| Norway | Oslo | Oslo University Hospital | Urology | Catherine | Elizabeth | Philps Pereira |
|  |  |  |  | Kristin |  | Rennesund |
| Oman | Sohar | Sohar Hospital | Urology | Emad Eldin |  | Mousa |
| Pakistan | Faisalabad | Allied Hospital/Punjab Medical College and Affiliated Hospitals, Faisalabad | Department of Urology and Kidney Transplantation | Muhammad | Sheraz | Javed |
|  | Pakpattan | City hospital | Urology /Surgery | Kamran | Hassan | Bhatti |
| Philippines | Quezon | East Avenue Medical Center | Urology | Fidel Tomas | Moyano | Manalaysay |
| Poland | Wrocław | 4-th Military Clinical Hospital | Clinical Department of Urology | Wojciech |  | Panek |
| Portugal | Amadora | Hospital Professor Doutor Fernando da Fonseca | Urology | André |  | Barcelos |
|  | Braga | Hospital de Braga | Serviço de Urologia | Sónia | Afonso | Ramos |
|  | Coimbra | Centro Hospitalar e Universitário de Coimbra | Urologia e Transplantação Renal | João | Pedroso | Lima |
|  | Lisboa | Hospital de Egas Moniz | Urology | Rita | Rodrigues | Fonseca |
|  |  | Hospital Santa Maria | Departamento de Urologia | Tiago | Manuel | Ribeiro de Oliveira |
|  | Loures | Hospital Beatriz Ângelo | Urology | Luísa | Jerónimo | Alves |
|  | Porto | Centro Hospitalar do Porto | Urology | Bernardo | Lobão | Teixeira |
|  |  | Instituto Português de Oncologia do Porto Francisco Gentil, EPE (IPO-Porto) | Clínica de Urologia | João | Nuno | Pereira |
|  | Santarem | Hospital de Santarém | Serviço de Urologia | David | José Simões | Castelo |
|  | Viana do Castelo | ULSAM - Hospital de Viana do Castelo | Urology | José | Pedro | Cadilhe |
|  | Vila Nova de Gaia | Centro Hospitalar de Vila Nova de Gaia / Espinho | Serviço de Urologia | Pedro | Daniel Oliveira Rocha | Costa |
| Republic of Korea | Bucheon | Soonchunhyang University Bucheon Hospital | Urology | Woong Bin |  | Kim |
|  | Cheonan | Dankook University Hospital | Urology | Yumi |  | Seo |
|  | Daegu | Daegu Catholic University Medical Center | Urology | Hyun Jin |  | Jung |
|  | Daejeon | Chungnam National University Hospital | Urology | Seung woo |  | Yang |
|  |  |  |  | Yong-Gil |  | Na |
|  | Hwasun-gun | Chonnam National University Hwasun Hospital | Urology | Eu Chang |  | Hwang |
|  | Seoul | National Police Hospital | Urology | Seung Ki |  | Min |
|  | Suwon | St. Vincent's Hospital, The Catholic University of Korea | Urology | Hyunsop |  | Choe |
|  | Uijeongbu-si | The Catholic University of Korea | Urology | Sang Rak |  | Bae |
|  | Yangsan-si | Pusan National University Yangsan Hospital | Urology | Seungsoo |  | Lee |
| Romania | Bucharest | Fundeni Clinical Institute | Center for Uronephrology and Renal Transplantation | Alexandru | Iulian | Iordache |
|  | Iasi | DR. C.I.Parhon Clinical Hospital | Urological Clinic | Viorel | Dragos | Radu |
|  | Miercurea Ciuc | County Hospital | Urology | Singeorzan |  | Dorin |
|  | Sibiu | Sibiu Academic Emergency Hospital | Urology | Adrian | Gheorghe | Hasegan |
|  |  | Sibiu Academic Emergency Hospital | Urology | Mircea | Valentin | Pirvut |
| Russian Federation | Ivanovo | Ivanovo Regional Clinical Hospital | Urology | Alexey |  | Shevyrin |
|  | Moscow | European Medical Center | Urology | Natalia |  | Sumerova |
|  |  | Moscow city Vorokhobov’s hospital 67 | Urology | Pisarev |  | Sergey |
|  |  | N.I.Pirogov "National Medical-Surgical Center" DoH Russia | Urology | Yuriy |  | Brook |
|  |  | S.P.Botkin Clinical Hospital | Urology | Liubov |  | Sinyakova |
|  |  | S.R. Urology Institute | Infection and Inflammation | Tamara | Sergeevna | Perepanova |
|  | Novosibirsk | Novosibirsk Scientific Research Institute of Tuberculosis of Minzdrav | Urology | Sergey |  | Shevchenko |
| Serbia | Belgrade | Clinic of Urology | Intensive care | Djordje | Petar | Nale |
|  | Kraljevo | Studenia | Urology | Vladica | Branislav | Slavic |
| Singapore | Singapore | National University Hospital Singapore | Urology | Fiona Mei Wen |  | Wu |
|  |  |  |  | Edmund |  | Chiong |
| Slovenia | Ljubljana | University Medical Centre Ljubljana | Urology | Milena |  | Taskovska |
| Spain | Alcala de Henares | Hospital Universitario Principe de Asturias | Urology | Joanne | Slyth | Serrano Uribe |
|  |  |  |  | Nelson |  | Morales |
|  | Aviles | San Augustin University Hospital | Urology | Serenella |  | Monagas |
|  | Boadilla del Monte | Hospital HM Montepríncipe | Urology | José |  | Medina Polo |
|  | Don Benito | Hospial Don Benito Villanueva | Andres Lopez de Alda | Andres |  | Lopez de Alda |
|  | Elche-Alicante | Hospital General Universitario de Elche | Urology | Liliana |  | Garces |
|  | Fuenlabrada | Hospital Universitario de Fuenlabrada | Servicio de Urología | Hugo | Otaola | Arca |
|  | L'Hospitalet de Llobregat | Hospital Universitari de Bellvitge | Urology | Maria |  | Fiol Riera |
|  | Madrid | Hospital Central de la Defensa Gómez Ulla | Urology | Pablo |  | Conde Caturla |
|  |  | Hospital General Universitario Gregorio Marañón | Urology | Javier |  | Mayor de Castro |
|  |  | Hospital La Zarzuela | Urology | Iñigo |  | López Díez |
|  |  | Hospital Univeristario 12 de Octubre | Urology | José |  | Medina-Polo |
|  |  |  |  | Alejandro |  | González-Díaz |
|  |  | Hospital Universitario La Paz | Urology | Mario |  | Alvarez-Maestro |
|  | Manresa | Xarxa Assistencial i Universitària de Manresa | Urology | Laia |  | Sos |
|  | Murcia | Hospital Clínico Universitario Virgen de la Arrixaca | Urology | Pedro |  | López Cubillana |
|  |  |  |  | Alicia |  | López Abad |
|  | Palma | Hospital Universitari Son Espases | Servicio de Urología | Jose Luis |  | Bauza Quetglas |
|  | Salamanca | UNIVERSITY HOSPITAL OF SALAMANCA | Urology | Maria-Fernanda |  | Lorenzo-Gomez |
|  | SC de Tenerife | Hospital Universitario Nuestra Señora de Candelaria | Urology | Begoña |  | Ballesta |
|  |  |  |  | Sergio |  | Fumero Arteaga |
|  | Valencia | Hospital de Manises | Urology | Gonzalo |  | Garcia Fadrique |
|  | Valladolid | Hospital Universitario Río Hortega | Urology | Ana-María |  | Tapia Herrero |
|  |  |  |  | Ana María |  | Tapia Herrero |
|  | Vitoria-Gasteiz | Hospital Universitario Araba | Urology | Josep | M. | Campa |
| Sweden | Göteborg | Sahlgrenska | Urology | Mohammed | Adel M. | Alfawzan |
|  | Helsingborg | Helsingborg Hospital | Urology | Andreas |  | Forsvall |
|  | Linköping | Linköping University Hospital | Urology | Martin |  | Holmbom |
|  | Örebro | University Hospital Örebro | Urology | Ahmad |  | Al-Ghraoui |
|  | Skövde | Skaraborgs Hospital | Urology | Erik |  | Sagen |
|  | Sundsvall | Sundsvall Hospital | Urology | Johan |  | Styrke |
| Switzerland | Bellinzona | San Giovanni | Urology | Matteo |  | Ferrari |
| Tunisia | Nabeul | Mohamed Tahar Mâamouri University Hospital | Urology | Sataa |  | Sallami |
|  | Sousse | Teaching Hospital Sahloul | Urology | Tlili |  | Ghassen |
|  | Tunis | Hôpital Charles Nicolle | Urology | Skander |  | Zouari |
| Turkey | Ankara | Gülhane Research and Training Hospital | Urology | Selcuk |  | Sarikaya |
|  |  |  |  | Mustafa |  | Vurucu |
|  | Bayburt | Bayburt State Hospital | Urology | Anil |  | Erkan |
|  | Diyarbakir | Dicle University | Infectious Diseases | Recep |  | Tekin |
|  | Edirne | Trakya University Faculty of Medicine | Balkan Campus | Serdar |  | Madendere |
|  | Isparta | Süleyman Demirel University Training hostital | Urology | Taylan |  | Oksay |
|  | Istanbul | Bağcılar Training & Research Hospital | Urology | Serkan |  | Gönültaş |
|  |  | Bakirkoy Dr.Sadi Konuk Training And Research Hospital | Urology | Mithat |  | Ekşi |
|  |  | Hisar Intercontinental Hospital | Urology | Ersan |  | Arda |
|  |  |  |  | Basri |  | Cakiroglu |
|  |  | Istanbul Training and Research Hospital | Urology | Mustafa |  | Kadihasanoglu |
|  | Kayseri | Kayseri City Hospital | Urology | Gokhan |  | Sonmez |
|  | Samsun | Gazi State Hospital | Urology | Mustafa | Suat | Bolat |
|  | Sanliurfa | Harran University Faculty of Medicine | Urology | Bulent |  | Kati |
|  |  |  |  | Eyyup | Sabri | Pelit |
|  | Zonguldak | Bülent Ecevit Univercity Hospital | Urology | Reha |  | Girgin |
| Ukraine | Kyiv | SI "Institute of urology NAMS of Ukraine | 1-st Urology Department | Aleksandr | V. | Shulyak |
|  | Lviv | 5-th City State Hospital | Urology | Khrystyna | Vladimirovna | Yanovska |
| United Arab Emirates | Abu Dhabi | Franco-Emirien Hospital | Urology & Andrology | Medhat | Ahmed Mohamed | Elsayed |
|  |  | Franco-Emirien Hospital | Urology & Andrology | Medhat | Ahmed Mohamed | Elsayed |
|  |  | Mediclinic Airport Road | Surgery/Urology | Mahmoud |  | Alnesr |
|  | Dubai | Dubai Hospital | Urology | Mohammad | Hassan | Alhamad |
|  |  |  |  | Abdulmunem | Mohammed | Al Sadi |
| United Kingdom | Barnstaple | North Devon District Hospital | Urology | Soumya |  | Misra |
|  | Cheltenham | Cheltenham General Hospital | Urology | Theodore |  | Birks |
|  | Edinburgh | Western General Hospital | Urology | Roland |  | Donat |
|  | London | Guy's Hospital | Urology Centre | Rick |  | Popert |
|  |  | North Middlesex University Hospitals NHS Trust | Urology | Niyukta |  | Thakare |
|  |  | Royal Free Hospital | Urology | Paras |  | Singh |
|  | Norwich | Norfolk and Norwich University Hospital | Urology | Sanchia |  | Goonewardene |
|  | Slough | Wexham Park Hospital | Urology | Elsayed |  | Desouky |
|  | Stoke-on-Trent | Royal Stoke University Hospital | Urology | Mark | Fraser | Saxby |
|  | Swansea | Morriston Hospital | Urology | Gokul Vignesh |  | KandaSwamy |
|  | Swindon | Great Western Hospital | Urology | Haitham |  | Abdelmoteleb |
| Vietnam | Ho Chi Minh City | Bihn Dan | Urology A | Chuyen | Le | Vu |
|  |  |  |  | Doan | Huu | Pham |
|  |  |  |  | Vinh Hung |  | Tran |
