# Supplementary figures and images for: The negative aftermath of prostate biopsy: prophylaxis, complications and antimicrobial stewardship: results of the global prevalence study of infections in urology 2010–2019
Source: World J Urol. 2021 Feb 22;39(9):3423–32. doi: 10.1007/s00345-021-03614-8 (PMC8510929; doi:10.1007/s00345-021-03614-8)

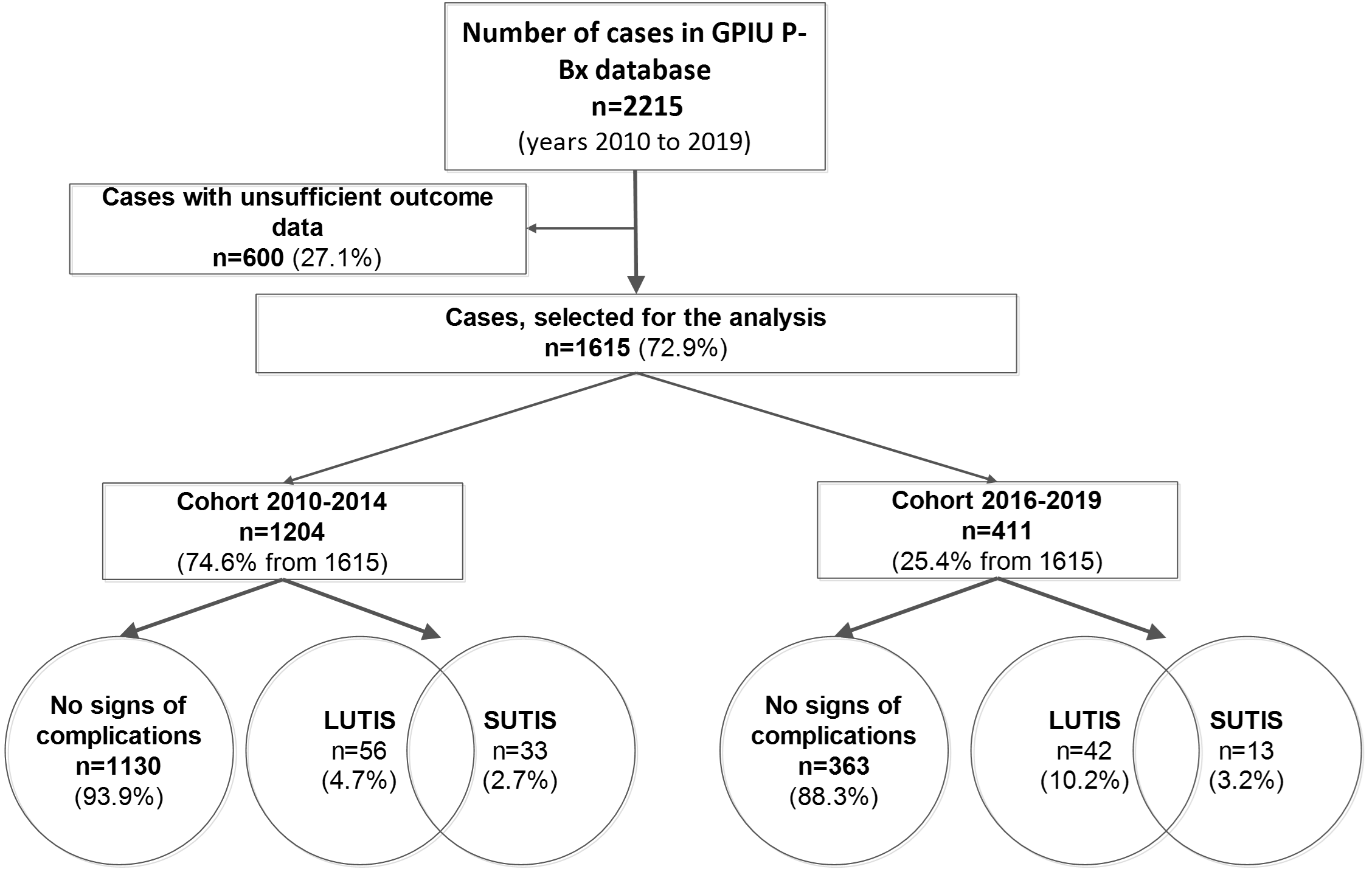

Supplement: Supplementary file 2 — Supplementary file2 Supplementary Figure 1. Flow chart of inclusion and outcomes (TIF 321 KB) [file 345_2021_3614_MOESM2_ESM.tif]

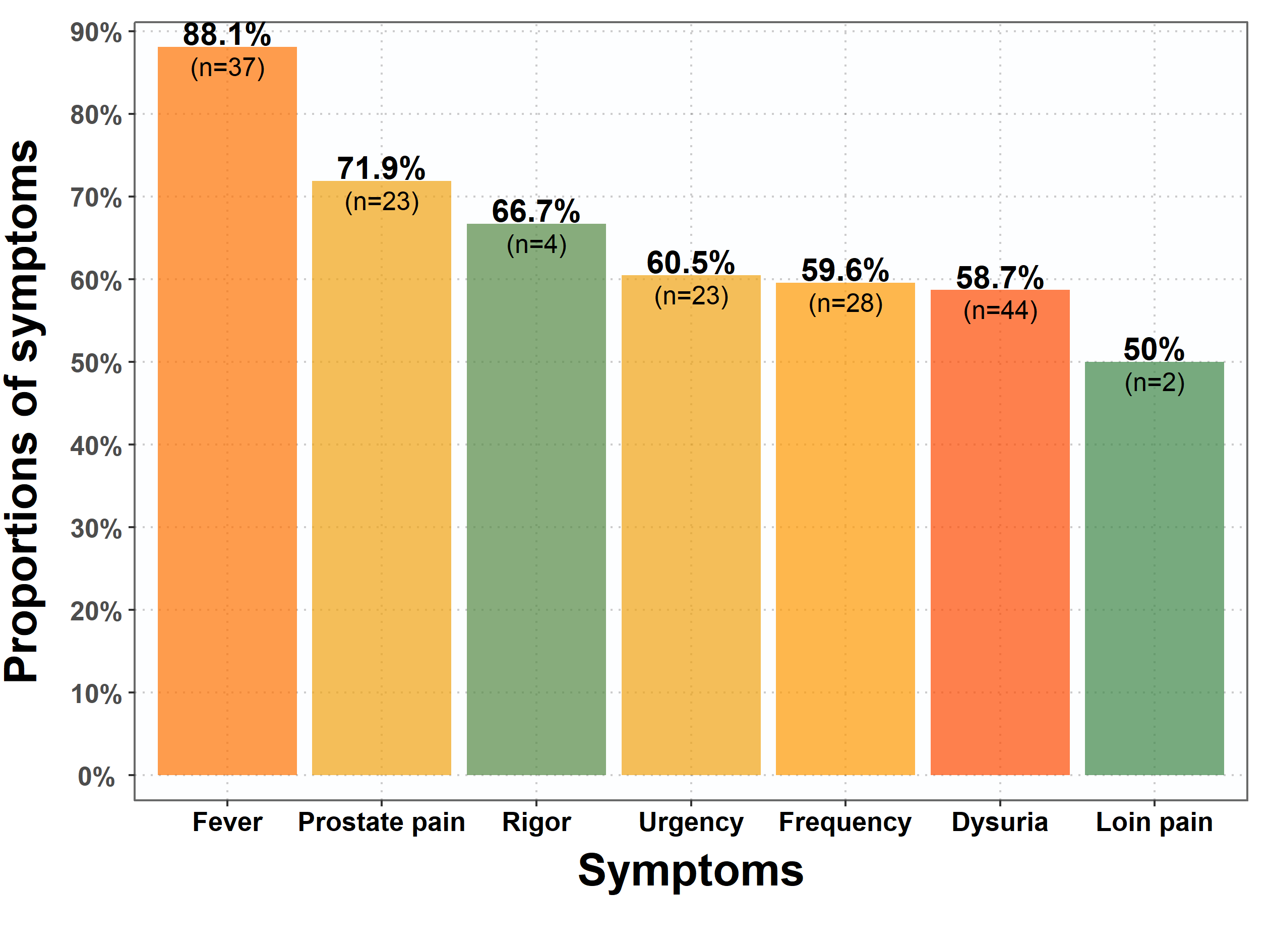

Supplement: Supplementary file 3 — Supplementary file3 Supplementary Figure 2. Proportions of symptoms leading to antimicrobial treatment within 2 weeks after P-Bx (PNG 109 KB) [file 345_2021_3614_MOESM3_ESM.png]
